# Illusions of Imagery and Magical Experiences

**DOI:** 10.1177/2041669519865284

**Published:** 2019-08-15

**Authors:** Vebjørn Ekroll

**Affiliations:** Department of Psychosocial Science, University of Bergen, Norway

**Keywords:** visual imagery, mental simulation, topology, magic, metacognition, attribute substitution

## Abstract

In recent years, there has been a growing interest in the idea that we may gain new insights in cognitive science by studying the art of magic. Here, I offer a first exploratory overview and preliminary conceptual analysis of a class of magic tricks, which has been largely neglected in this pursuit, namely, a set of tricks that can be loosely defined as topological tricks. The deceptive powers of many of these tricks are difficult to understand in light of known psychological principles, which suggests that closer scientific scrutiny may raise interesting questions and challenges for cognitive science. I discuss a number of known and novel psychological principles that may explain why these tricks evoke the strong feelings of impossibility that are characteristic of magical experiences. A profound and detailed understanding of how topological tricks evoke magical experiences remains elusive, though, and more research on this topic could advance our understanding of perception, imagery and reasoning.

The idea that we may advance cognitive science by studying what magicians do and why it works has gained considerable traction in recent years (Kuhn, 2019; Kuhn, Amlani, & Rensink, 2008; Macknik et al., 2008; Rensink & Kuhn, 2015). Establishing links between the art of conjuring and cognitive science appears to be an interesting and rewarding exercise for several reasons. First, as a tool for teaching, magic tricks can be used as particularly powerful demonstrations of well-known theoretical principles and phenomena in cognitive science, such as inattentional blindness (Kuhn & Tatler, 2011) or Gestalt principles of perceptual organization (Barnhart, 2010; Ekroll, Sayim, & Wagemans, 2017). Second, the academic study of magic may guide us toward questions and topics that have been largely neglected in mainstream cognitive science (Rozin, 2006), such as the nature of magical experiences and the sense of wonder accompanying them (Lamont, 2017; Leddington, 2016; Rensink & Kuhn, 2015). Third, the study of magic may provide the opportunity to discover cognitive processes that are still poorly understood (Thomas, Didierjean, Maquestiaux, & Gygax, 2015). As an example, Ekroll et al.’s (2017) analysis of the role of amodal completion in magic led to the discovery of an intriguing illusion of absence (Øhrn, Svalebjørg, Andersen, Ring, & Ekroll, 2019; Svalebjørg, Øhrn, & Ekroll, 2018) that was previously unknown in cognitive science. In this vein, the art of magic can serve as a touchstone for cognitive science: If there are magic tricks which we cannot understand based on the theoretical machinery provided by cognitive science, we obviously need to expand or revise our theories.

## What Does It Mean to Understand a Magic Trick?

**Figure 1.**
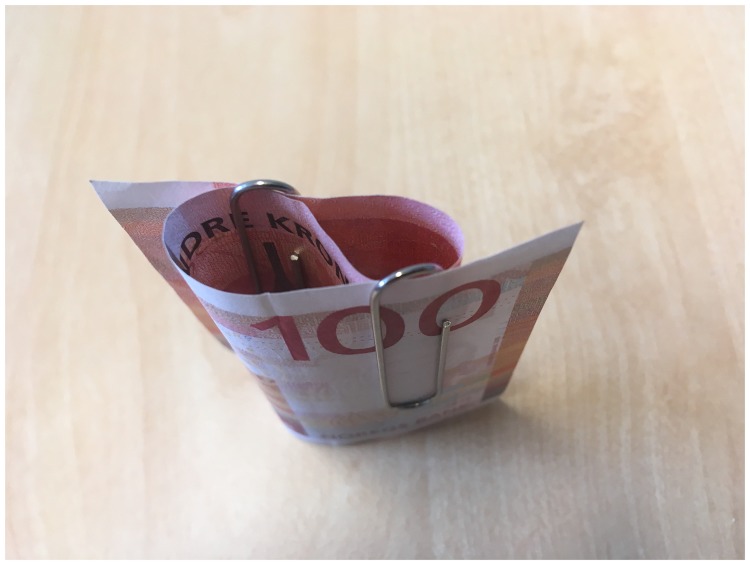
To perform the linking paper clips trick, fold up a bank note (one third of it in one direction, one third in the other) and attach the opposite folds to the ends as shown in the figure. When you pull the banknote straight, the paper clips jump off and automatically become linked, as if by magic.

Although several magicians have developed sophisticated theoretical accounts of the nature and art of magic (Ortiz, 2006; Tamariz, 1988), the art of magic is first and foremost an applied field. Since knowing how to do something is not necessarily the same as understanding it, it is therefore likely that the art of magic includes tricks that are not very well understood, even by magicians. To appreciate this point, you may want to perform the linking paper clips trick (Wilson, 1988), which is illustrated in Figure 1 and performed in Movie 1. A banknote is folded twice and a paperclip is placed over each of the two opposing folds in the bill (Figure 1). When you pull the bill straight by its ends, the paperclips jump off and—as if by magic—suddenly have become linked. You probably won’t feel like you really understand how this happened even after having tried the trick out for yourself. For some reason, people seem to regard it as impossible that just pulling at the ends of the banknote will link the two paperclips, but it is not obvious what that reason is. This example illustrates that one may be able to perform a magic trick without having a clear understanding or “mental image” of what is really going on, let alone why people experience the trick as magical (i.e., impossible). From the perspective of cognitive science, the magic trick can only be said to be completely understood if we can specify why people experience it as impossible (and hence magical, see Ortiz, 1994).

## Mapping Tricks Onto Psychological Principles

To assess to what extent the plethora of tricks and effects used by magicians can be accounted for based on extant theories and known principles in cognitive science, it seems sensible to consider to what extent it is possible to develop a taxonomy of magic tricks that maps them onto known underlying psychological principles. Kuhn, Caffaratti, Teszka, and Rensink (2014) have initiated such a systematic effort and proposed a preliminary psychologically based taxonomy of misdirection.

## Aims and Plan of the Study

In this study, I aim to contribute to the development of a more complete taxonomy mapping tricks onto underlying psychological principles by considering an interesting domain of magic that thus far has been largely neglected in psychological research. The type of tricks I shall consider can be loosely described as topological tricks, because they involve flexible materials such as paper, cloth, rope, and rubber bands (Gardner, 1956). An informal observation spurring my interest in this kind of tricks is that although they are often quite simple and self-working (Fulves, 1981, 1990), they nevertheless tend to be surprisingly robust and powerful, which suggests that they may rest on hitherto unknown or underestimated cognitive phenomena (Ekroll et al., 2017).

A broad preliminary working hypothesis is that this type of magic tricks works so well because it exploits our limited mental capacities for representing flexible objects and mentally simulating how they might be deformed. My foray into the psychology of topological magic is preliminary. Where possible, I try to formulate some basic overarching principles that may help explain how various topological tricks work, but there are many loose ends, where I can only point to interesting challenges for future research and raise interest for this intriguing field of inquiry.

**Movie 1.** The linking paper clips trick.

## Knots as Perceptual Mess

**Figure 2.**
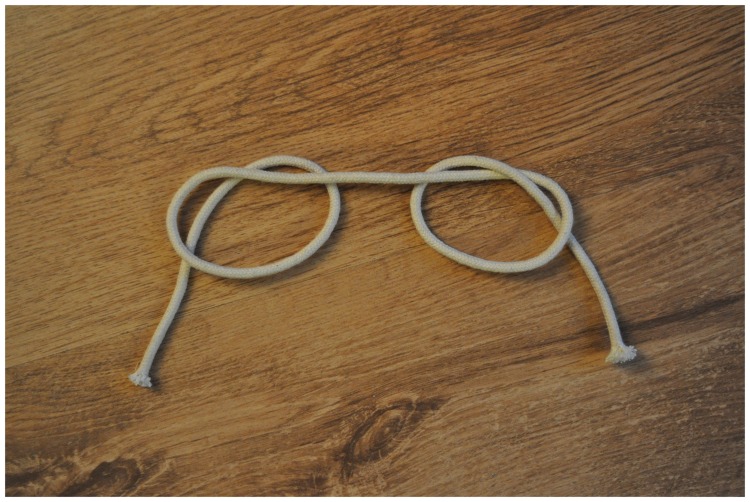
A real and a fake overhand knot. Note how difficult it is to tell which one is the real knot without further scrutiny.

Consider the disappearing knot trick demonstrated and revealed in Movie 2. Here, the magician seems to tie an overhand knot that magically disappears when the magician pulls on the ends of the rope. The simple secret behind the trick is that the magician does not tie the overhand knot in the first place. Rather, with the help of some thin invisible thread tied around a bend in the rope, the rope is coiled up such that it looks similar to an overhand knot. When the magician pulls on the rope, the invisible thread breaks, and the coiled-up rope simply straightens, but it looks like a knot magically disappears. Figure 2 shows both the fake knot and the real one. Note how difficult it is to see which knot is the real one “just by looking.” Although one has the impression of seeing each “knot” with utmost clarity, it requires quite some mental effort to determine their three-dimensional (3D) spatial layout well enough to be able to tell which of them is the real knot. One has to mentally trace the rope in a “serial” fashion and apply mental effort to make the distinction.

**Figure 3.**
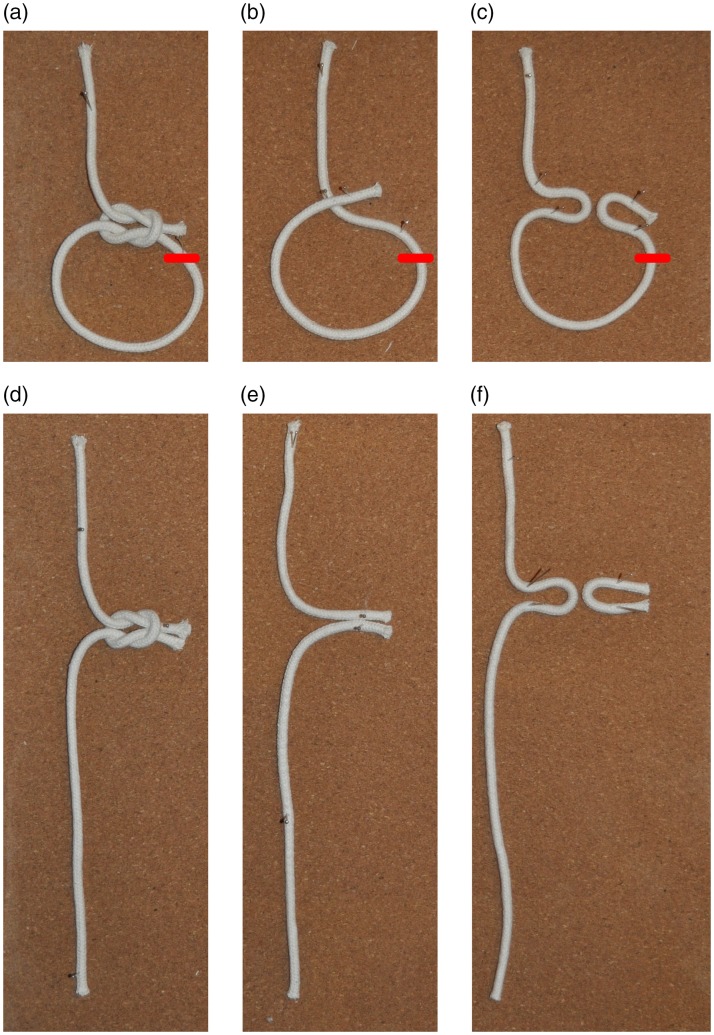
In the version of the cut-and-restored rope trick described in Cassidy and Stround (1989, p. 64), a loop is tied as in Panel a, and the rope is cut at the location indicated by the red bar. The rope is held at the top, and when the loop is cut, the rope hangs down as in Panel d. Panels b and e illustrate the perceived correspondences, while Panels c and f illustrate the actual correspondences.

**Figure 4.**
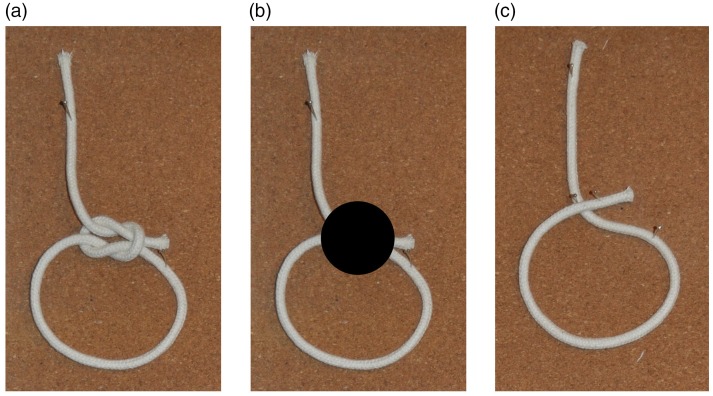
Note how the knot in the cut-and-restored rope trick (a) and amodal completion behind an occluder based on the Gestalt principle of good continuation (b) both lead to the same wrong impression of the basic structure of the rope (c). The knot itself has no influence on how we perceive the structure of the rope; it is as though we were effectively blind to it if we do not engage in effortful mental curve tracing.

A similar point is demonstrated by the version of the cut-and-restored rope routine shown in Movie 3 (Cassidy & Stroud, 1989; first described in Tarbell, 1942, as “Ted Collins’ Panama Rope Mystery”). Here, the magician first ties a square knot in the rope, thus producing a loop with a somewhat longer loose end (Figure 3(a)). He then proceeds to cut the loop at a point close to the knot (short red line). The result of this is shown in Figure 3(d). He then makes the knot magically pop off the rope simply by pulling on the upper and lower ends.^1^ This trick tends to produce a strong magical effect, but it is essentially self-working. The basis of the trick seems to be that the spectator automatically establishes false correspondences between the different parts of the rope leading “into” and “out of” the knot. In Figure 3, the middle column illustrates the perceived correspondences, while the right-hand column illustrates the actual correspondences. Due to this confusion, the spectators have the impression that the magician cuts the rope approximately in the middle, while in fact he is just cutting off a small piece knotted around the unsevered main part of the original rope. It is interesting to note that—as illustrated in Figure 4—the erroneous correspondences made by the spectators are exactly the same as those that would occur based on the Gestalt principle of good continuation (Wertheimer, 1923) if the knot were hidden from direct view by a small occluder (Barnhart, 2010; Ekroll et al., 2017). Thus, although the knot is readily visible, the spectator behaves as if it were not.

**Movie 2.** The disappearing knot trick.trick

The basic phenomenon illustrated by these two tricks (and many others as well) is reminiscent of, and probably related to, several well-known but poorly understood phenomena of visual awareness. In the first instance, it is reminiscent of systematic limitations in the visual perception of spatial relationships and topology (Koenderink, 1984; Minsky & Papert, 1969; Ullman, 1984), where determining the topological properties of moderately complicated curves requires effortful and “serial” mental curve tracing (Jolicoeur, Ullman, & Mackay, 1986). On a more general level, the mental experiences evoked by these knot tricks are reminiscent of those associated with change blindness, inattentional blindness, and peripheral vision in the sense that they involve a failure of visual meta-cognition (or perhaps more accurately “meta-perception”), that is, a discrepancy between what we actually perceive and what we intuitively believe that we are able to perceive (Levin, 2002): Although we have the impression that we experience the stimuli with utmost clarity, our objective performance in “seeing” relatively simple spatial and topological relationships is quite poor. Although the overhand knot involved in the first of the two tricks considered above is just about the simplest knot there is, our ability to discriminate it from a fake knot seems to be surprisingly limited. Considering this, it is tempting to speak of “topology blindness,” in loose analogy to “change blindness.” It seems to be the case that we do not see knots as well-defined 3D objects but rather as undifferentiated and perceptually unorganized entities—or simply as “perceptual mess” (see Casati, 2013, for a related observation). It has been argued that failures of meta-cognition are key factors in enabling magicians to create strong magic (Ekroll et al., 2017; Kuhn, 2019; Kuhn et al., 2014). Given that change blindness and inattentional blindness involve a failure of visual meta-cognition, this would explain why attentional misdirection is such a widely used and potent factor in magic (Ortega, Montañes, Barnhart, & Kuhn, 2018). Ekroll et al. (2017) have argued that the well-known perceptual phenomenon of amodal completion (Van Lier & Gerbino, 2015) also involves a failure of visual meta-cognition and that this explains the surprising potency of the many tricks that are based on amodal completion. In line with this reasoning, I propose that there is an analogous failure of visual meta-cognition in the perception of knots, which also makes them excellent tools for creating strong magic. Here, the failure of visual meta-cognition is that we visually experience knots as clear and distinct although our perceptual system merely fails to represent them in any detail and instead treat them as undifferentiated and unorganized “perceptual mess.”

**Movie 3.** The cut and restored rope trick.

## Illusions and Limitations in Visual Imagery and Mental Simulations

Visuospatial imagery has been argued to play a central role in human thinking. According to Shepard (1978), imagining objects and their transformations in space makes it possible to explore many possibilities without having to carry out the operations out in physical reality. While there is little doubt that the ability to perform mental simulations (Hegarty, 2004; Moulton & Kosslyn, 2009) is a central and useful feature of human cognition, the nature of the mental representations on which these simulations operate have been the topic of heated debate (Chambers & Reisberg, 1985; Pylyshyn, 1973, 2003b; Reed, 1974). A major stone of contention in the so-called imagery debate (Kosslyn & Kosslyn, 1996; Pylyshyn, 1981) was whether the underlying representations are essentially pictorial or propositional in nature. To gain a better understanding of the representations and mechanisms underlying this kind of mental simulation, it is particularly instructive to consider cases where the mental simulations seem to consistently yield misleading intuitions (Hinton, 1979; Margolis, 1998a, 1998b; Pearson, 1998; Pearson & Logie, 2015; Pylyshyn, 2003a). From this perspective, the study of topological magic tricks seems particularly interesting, because it seems to be replete with such “illusions of imagery.” In this section, I describe a number of illustrative examples.

Since any single trick may involve several psychological principles, and the same psychological principle may be involved in several tricks, I shall begin by merely describing the tricks in this section, to set the stage for a more systematic discussion of potential unique and shared underlying psychological principles in the following sections.

**Movie 4.** The Houdini chain shackle escape trick.

### The Houdini Chain Shackle Escape

**Figure 5.**
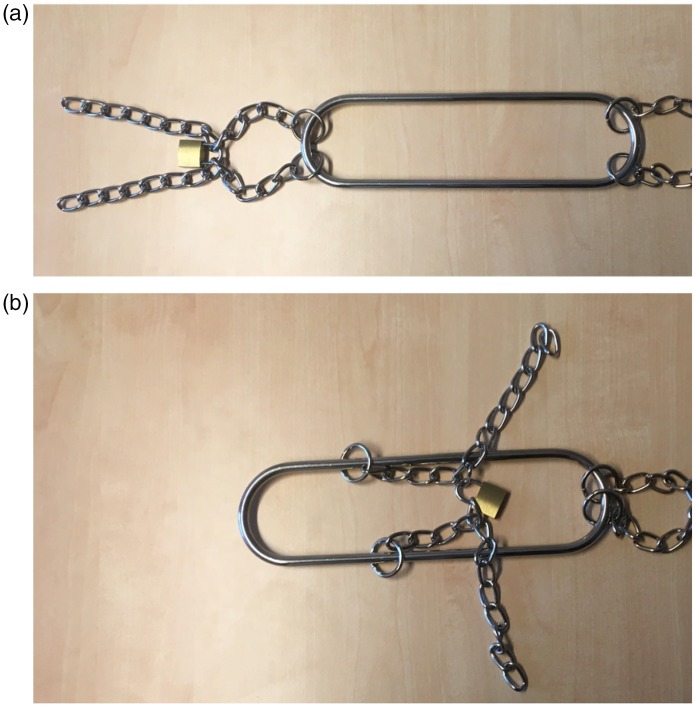
In the Houdini chain shackle release trick, the hands of the magician are chained to the outside of an elongated metal frame. That is, the hands are held in the two loops of chain shown in (a). By twisting the wrists and the loop around the frame, the chain can be made to slide along it (b), which provides a large opening from which the hand is easily pulled out.

In this trick, the magician’s hands are tied with chains to an elongated metal frame with rounded ends (see Movie 4). Although it appears impossible for the magician to sneak his hands out of the tightly fitted chains, it is actually quite easy: When one of the hands is rotated into the frame, the chains can slide along the frame to create a big opening (see Figure 5 and Movie 5). The interesting question is why it is so difficult to imagine this.

### The Afghan Bands

**Figure 6.**
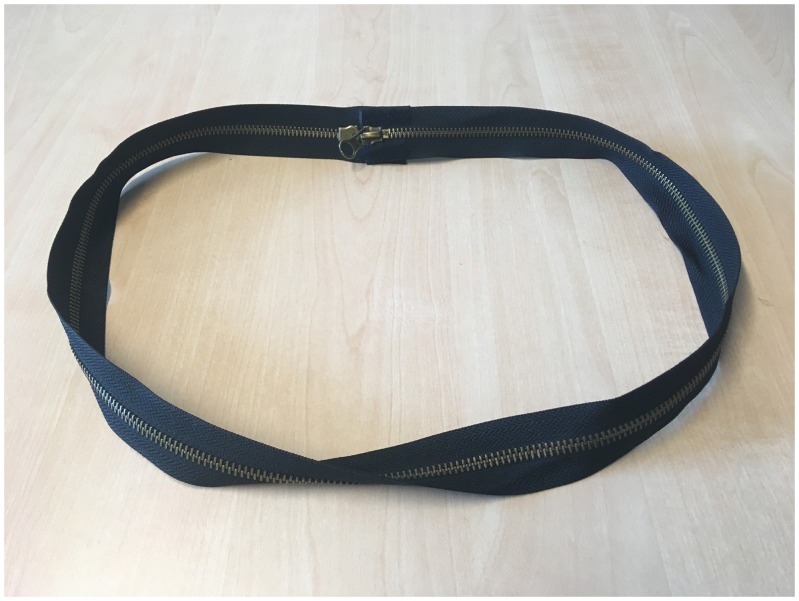
A Möbius band made of a zipper. The two ends are joined to form a loop, but before that, one of the ends is twisted by 180°. If the Möbius band is divided along the middle (i.e., by unzipping the zipper), a single long loop is obtained. People tend to be surprised by this, expecting instead to obtain two loops. It is easier to understand that a single loop must indeed result by thinking of the band as two adjacent strips. Due to the 180° twist, each of the ends of one of the thin strips is attached to one of the ends of the other one, so that a single long loop results.

In this trick (Wilson, 1988, see also Movie 6), a loop obtained by joining the two ends of a long strip is cut along the circumference (in the Movie, a zipper is used to “cut” the loop, see Figure 6). This is done 3 times, one after the other, and each time, a different result is obtained. In the first case, two separate loops are obtained, as one would expect. In the second case, however, they end up as a single loop which is twice as long. In the third case, two loops are obtained again, but they are linked to each other. The secret difference between the three loops is that the strips they are made of have been twisted a different number of times before they were joined together to form a loop. The first loop has not been twisted, the second has been twisted by half a turn (180°), and the third has been twisted by a complete turn (360°). The different amounts of twist are not easily noticed when the loops are long. Even if the spectators know about the different amounts of twist, however, the outcomes obtained with the second and third loops are probably deeply counterintuitive. The loop that has been twisted by half a turn is the Möbius band (Figure 6), which is well known in topology (Hilbert & Cohn-Vossen, 1952; Pickover, 2006), but also notoriously difficult to get one’s head around. It seems very difficult to imagine that cutting the Möbius strip all around along the middle of the strip fails to produce two separate objects. The interesting question is why this is so difficult.

**Movie 5.** The secret behind the Houdini chain shackle escapte trick.

**Movie 6.** The Afghan bands routine performed using a zipper.

### The Handcuffs Puzzle

**Figure 7.**
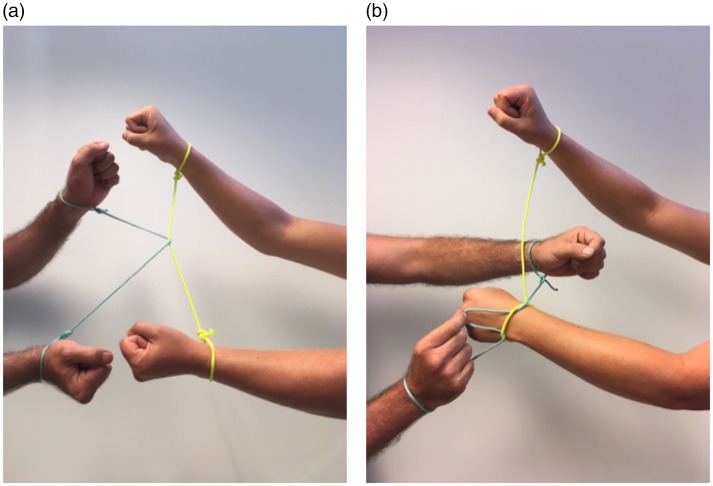
(a) In the handcuffs puzzle, the hands of two participants are handcuffed with a piece of rope, such that the participants are tied together. The task of the participants is to release themselves from each other without cutting the ropes or untying the knots. (b) The solution is to wrap the rope of one of the participants under the loop and over the hand of the other participants.

This is more of a party game (Gardner, 1956) than a magic trick, but the same basic principle is used in many magic tricks such as the *Telekinetic Ring* (Fulves, 1990, p. 130) or *Locked in Place* (Fulves, 1990). Each of two participants is handcuffed with a piece of rope, where loops are tied around both wrists (Figure 7(a)), and the handcuffs are looped around each other, such that the participants are linked to each other. The task of the participants is to free themselves from each other without cutting the ropes or untying the knots fixing the loops around each wrist. This party game makes for great fun, as the participants often assume hilarious poses when trying to free themselves from each other. It is very rare that participants who do not know the trick in advance are able to figure out the solution, which is to pull a loop of the rope of one of the participants into the loop around one of the wrists of the other participant and drag it around the hand before pulling it out on the other side (Figure 7(b)). Again, the interesting question is why it is so difficult to imagine this simple solution.

In a perhaps even more mind-bending variant of this puzzle, a longish loop of rope is tied around the arm of a person keeping his thumb in her vest pocket. The challenge is to remove the loop of rope without untying or cutting it or releasing the thumb from the vest pocket. Believe it or not, it is possible (Gardner, 1956). Indeed, it is also possible to take off your vest without removing your jacket (Gardner, 1956).

### Hart’s Link

**Figure 8.**
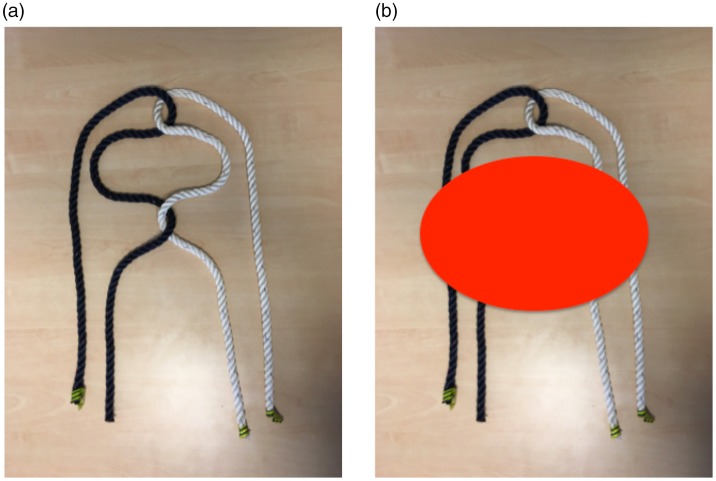
Illustration of the secret behind the routine called Hart’s link (see Movie 7). The magician twists the two ropes around each other as shown in Panel a but only shows the upper and lower parts of the ropes. The second turn of the ropes, which “undoes” the first remains hidden behind the magician’s head (the red disk in Panel b).

In this trick, the magician holds a rope folded up in the middle in each hand and points out that it is impossible to link the two loops to each other without threading the end of one rope around the other rope. He then takes his hands with the two unlinked loops behind his head and does just that, while the four ends remain in full view (Movie 7). The effect is stunning, but the method is disappointingly simple: The magician merely wraps one of the loops around the other rope, making sure that only half of the loop tied around the other rope is visible above the head (Figure 8). There are several variants of this trick (see, e.g., Fulves, 1981), some of which sell at exorbitant prices, demonstrating how hard it is to figure out the secret for oneself. Again, the interesting question is why the simple secret behind this trick is so difficult to figure out.

**Movie 7.** Hart's link.

### The Belt Trick

**Figure 9.**
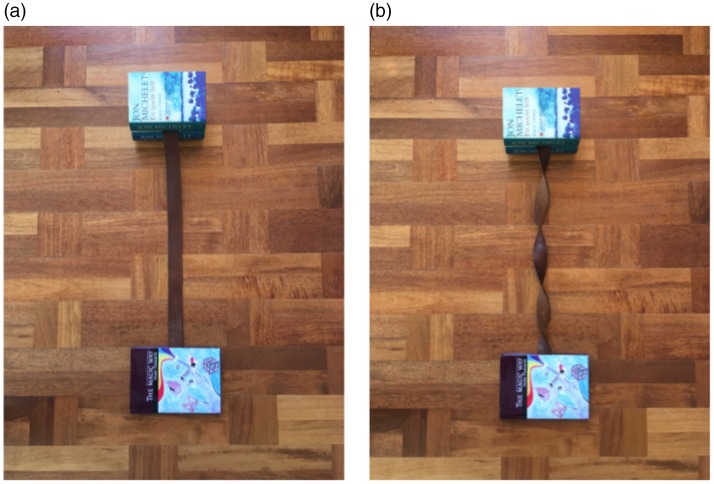
Illustration of the premises for the belt trick. If a straight belt (a) is twisted by two full turns (720°) to obtain the situation in (b), it is actually possible to untwist the belt without changing the orientation of either end of the belt. Here, the ends are highlighted by the books they are tucked into. Believe it or not, the twists disappear if you move the ends a bit closer to each other and wrap the middle of belt around one of them, as in Movie 8.

The belt trick (Kauffman, 2001; Pengelley & Ramras, 2017) and different variants of it, such as Dirac’s string trick or Feynman’s plate trick, are used in the teaching of physics to elucidate the rather counterintuitive behavior of elementary particles and the concept of half spin. Although it is not used by magicians, it tends to evoke the illusion of impossibility that is characteristic of magical experiences (Ortiz, 1994). Movie 8 shows a typical demonstration of this “magic trick which is not magic, but which reflects a fundamental yet little known property of the space in which we live” (Bolker, 1973, p. 984). Like in the movie, a physics professor may start with an untwisted belt (Figure 9(a)). Then, he goes on to twist it by two full turns (720°), obtaining the configuration in Figure 9(b) and proclaims that it is possible to untwist the belt again (returning to the state in (a)) without ever changing the orientation of either end of the belt (or the books in which the ends are fixed in Figure 9). Although most people would probably regard that as downright impossible, the professor shows that it is indeed possible, as shown in Movie 8. Interestingly, even when you know what to do to untwist the belt, and you see the whole process in front of your own eyes (try it out for yourself), it still feels quite counterintuitive or even impossible that the belt should end up being untwisted, although it is tangibly demonstrated that it is indeed possible. Again, the question is why this is so.

**Movie 8.** The belt trick.

### The Twisted Band

**Figure 10.**
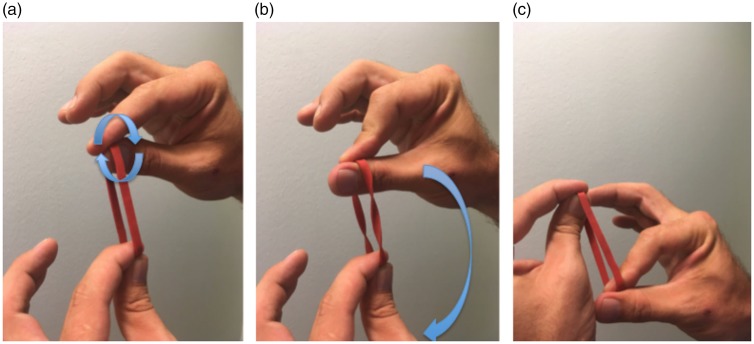
In the twisted rubber band trick, the prankster holds a rubber band pinched as shown in (a) and then twists the upper part of the rubber band by 360° by rolling it between his fingers. Thus, the two strands between the upper and lower parts are twisted by 360° (b). The rubber band is then handed over to the spectator, who is asked to hold it in exactly the same way. That is, he is also supposed to hold the upper part with his right hand and the lower part with his left hand. The challenge is to untwist the band without releasing or changing his grip on the rubber band. This is impossible. But when the rubber band is handed back to the magician, it can easily be untwisted by rotating the upper hand by 180° around the lower hand ((b) and (c)).

In this stunt (Gardner, 1956), the prankster starts by holding a rubber band as shown in Figure 10(a). He then twists the rubber band by 360° between his right-hand fingers, which leads to the situation depicted in Panel B. A volunteer is then asked to take the band from the prankster by grasping it in exactly the same manner (the volunteer take the top of the rubber band held by the prankster’s right-hand fingers with his own right-hand fingers, and the bottom with his left-hand fingers). The challenge is to remove the twists in the band without changing the grip on the two ends. The volunteer will find that this is impossible. But when the band is handed back to the prankster in exactly the same way, he easily removes the twists simply by pulling his right hand downwards (Panel C). The reason why this is possible for the prankster and not possible for the volunteer is that because the volunteer holds the band from the opposite sides, the direction of the twist is also opposite relative to the hands of the person who holds the band. If the volunteer were to make the same move as the prankster, he would not remove the twist but rather add a second one. When the prankster untwists the rubber band by the final move (Panel C), people tend to be surprised. Why is this so? Why is it so difficult to figure out that the orientation of the rubber band relative to the hands is key? As an additional point of interest, preliminary informal observations suggest that people find it rather counterintuitive that the move from Panels B to C, which can be thought of as rotating the upper hand by 180° around the lower hand (or vice versa) means that the band gets twisted by 360° (at each of the two strands, from top to bottom).

**Movie 9.** The four-dimensional hanks routine performed using drinking straws.

### The Four-Dimensional Hanks Routine

**Figure 11.**
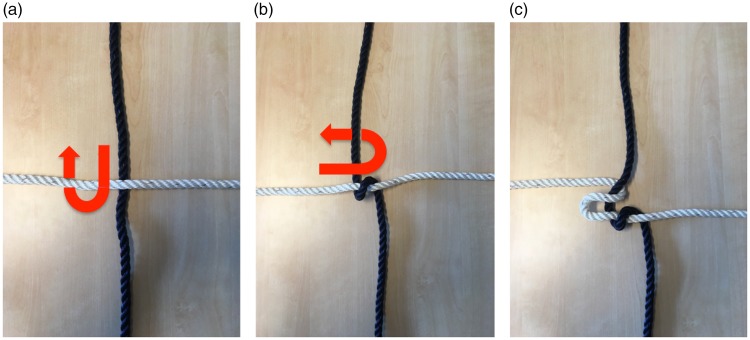
In the four-dimensional hanks routine (here illustrated with ropes), two pieces of rope are first positioned in a cross (a). The upper part of the vertical rope is looped once around the horizontal rope, resulting in the situation shown in (b). Then, the left-hand part of the horizontal rope is looped around the vertical rope, resulting in the situation shown in (c). Although one would think that the ropes are now knotted together by two loops, they are actually not tied at all, as closer scrutiny of the tangle in (c) reveals. This is because the second loop actually reverses the effect of the first one.

In this trick (Fulves, 1981, p. 85), two handkerchiefs (or other similar items, such as two pieces of rope or two drinking straws) are used (see (Movie 9). The vertical rope is first tied around the horizontal rope (Figure 11(a) and (b)) and then the latter is tied around the former (Figure 11(b) and (c)). When the magician pulls on the ropes, they seem to penetrate right through the “double knot.” The secret is that the second act of tying, rather than producing a second knot, simply has the effect of untying the first knot. This is rather counterintuitive, but closer scrutiny of the resulting “knot” (Figure 11(c)) shows that this is indeed the case. The interesting question is why this is so counterintuitive.

### The Jumping Rubber Band Trick

**Figure 12.**
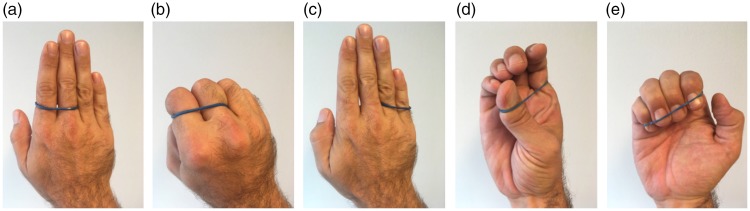
In the jumping rubber band trick, the magician puts a rubber band around his index and middle fingers (a). The fingers are closed into a fist (b), and when the fingers are stretched out again (c), the rubber band magically jumps from the index and middle fingers to the ring finger and the pinky. ((d) and (e)) To create this illusion, the magician uses his thumb, which is hidden from direct view, to create an opening through which all four fingertips are curled while the hand is closed to a fist. This implies that the index and middle finger, which are already in the loop, get out, while the ring finger and the pinky get in.

The jumping rubber band trick (Fulves, 1990; Wilson, 1988) is illustrated and explained in Figure 12. The magician starts with a rubber band around the index and the middle fingers (a). He then closes all the fingers to a fist (b), and when he extends the fingers again, the rubber band has magically jumped to the ring and pinky fingers (c). What the magician does—how could it be otherwise—is to get the two fingers already in the loop formed by the rubber band out of it and the other two fingers into it. Importantly, though, he does not get the two first fingers out of it by simply reversing the action of sticking them into it fingertips first—which would be to pull them out fingertips last. Rather, the fingers fold back ((d) and (e)) such that the fingertips leave the loop first.

### The Wholesale Ring Removal Trick

**Figure 13.**
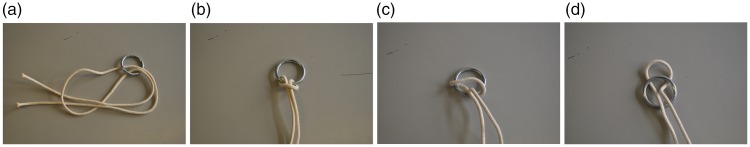
In the wholesale ring removal trick, a ring is tied to a piece of rope as shown in (a) and (b). The two loose ends of the rope are held by one of the spectators. Then, the magician covers the ring with a piece of cloth and releases the ring from the rope under the cover of the cloth. This appears impossible, but it is actually quite easy, as illustrated in (c) and (d).

In this trick (James, 1975), the magician ties a rope to a ring using a lark’s head knot (see Figure 13(a) and (b)). While the two loose ends of the rope are being held securely by a spectator and remain in plain view, the magician briefly covers the ring from the direct view (in his hand or under a handkerchief) and the ring immediately appears to have magically penetrated the rope, now being completely released from it. The simple secret behind this trick is that the ring is released from the rope by slipping the loop around the ring (Figure 13(c) and (d)).^2^ Why is it so difficult for the spectator to figure out how this is done?

**Movie 10.** The Gordian knot.

### The Gordian Knot

**Figure 14.**
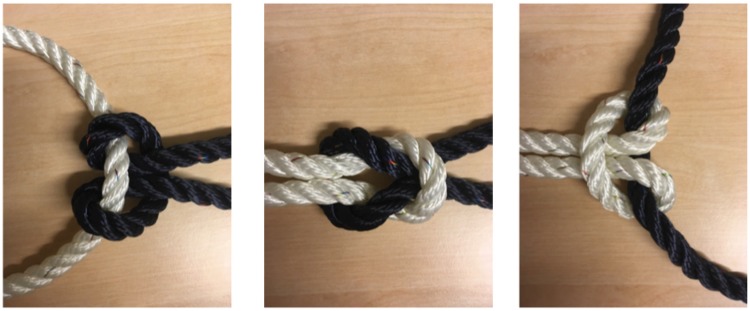
Illustration of the principle that underlies the Gordian knot routine. If one starts with the configuration in the middle, either of the configurations on the left or the right are immediately obtained by pulling one of the ropes straight. Viewing the configuration on the left and the right, it feels natural to say that one rope is tied around the other, but topologically speaking the situation is perfectly symmetrical, which is immediately apparent in the middle panel.

In this trick (Duraty, 2008, see Movie 10), the magician ties one rope around another one using a lark’s head knot. He then ties the second rope around the first one, using the same knot. When the magician pulls the ends of the ropes apart, they magically separate, as if they penetrate through the knots. Figure 14 illustrates why this seemingly impossible event happens. Although the first rope is tied around the other (left), the role of the two ropes in the knot is perfectly symmetrical (middle). If one starts with the configuration in the middle, either of the configurations on the left and the right is immediately obtained by pulling one of the ropes straight. Thus, the configurations on the left and the right are topologically identical, although they look very different. When the magician ties the second lark’s head knot, he is thus actually just untying the first one.

### Buttonholed

**Figure 15.**
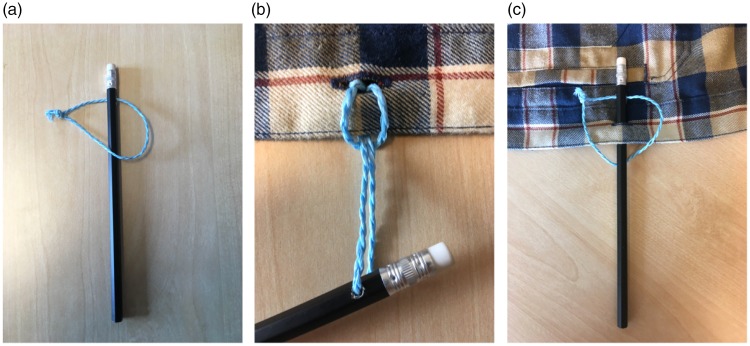
In the buttonholed stunt, a pencil with a loop of string attached to one end is used (a). Importantly, the loop is shorter than the pencil, such that it cannot be pulled over the long end. The prankster quickly ties the loop and the pencil to the buttonhole of the victim, resulting in the knot shown in (b). The victim will typically find it impossible to release the loop and the pen from the buttonhole. It is probably not so difficult to get from the situation in (b) to the situation in (c), but it appears that it is very difficult to realize how to get from the situation in (c) to the solution, which is to pull the piece of shirt where the buttonhole is through the loop until the buttonhole can be pulled off the long end of the pencil.

In this stunt invented by Sam Loyd (Fulves, 1981), a pencil with a loop of string attached to one end is used (Figure 15(a)). The loop is quickly tied to a buttonhole in the spectator’s shirt, resulting in the knot shown in (b). Now, the spectator is challenged to remove the loop and the pencil from the shirt. Typically, the spectator will find this impossible. It is not too difficult to get from (b) to (c). The difficulty seems to be to get from (c) to the solution, which is to pull the piece of the shirt with the buttonhole through the loop until the buttonhole reaches the other end of the stick and can be pulled of it. Why is it so difficult to see this?

### Coin Through Hole

In this old stunt (Einhorn, 2015), the challenge is to push a coin through a smaller circular hole in a piece of paper without tearing the paper. For instance, the task could be to push a nickel (diameter 21 mm) through a hole the size of a dime (18 mm). To most people, this would probably appear impossible, but it can easily be done by folding the piece of paper along a line which crosses the hole and then bend the folded paper such that the two opposite sides of the hole along the bend can get further apart without tearing the paper.^3^ Again, the question is why it is so difficult to come up with this simple solution.

## Toward a Theoretical Understanding of Topological Tricks

In the previous section, I have described and illustrated a number of different magic tricks, which can be loosely described as topological tricks in the sense of Gardner (1956) because they involve flexible materials. These tricks (and many related ones) seem particularly interesting because it is not obvious why they create the experience of impossibility (or magic) based on known principles from cognitive science. In the next section, I shall delineate some preliminary ideas about psychological principles which may be at play in these and related tricks. But before we get to that, some conceptual clarifications about (a) the notion of topological tricks, (b) the mapping of tricks to underlying psychological principles and (c) the relationship between magic and imagination may be in order.

### The Notion of Topological Tricks

While I believe that it is potentially interesting and useful to try to develop a notion of “topological tricks” that makes sense in terms of the underlying psychological principles, at least as a preliminary heuristic device, it is not straightforward to do so. My working definition of a topological trick is “a trick that is based, at least in part, on limitations, principles and heuristics in the mental processing of topological properties, such as connectedness and the possible transformations of bendable objects.” As pointed out by Gardner (1956), “the field of topological magic is restricted almost entirely to such flexible materials as paper, cloth, string, rope and rubber bands.” But it is worth noting that the converse is not true. Many tricks use such materials but are not based on principles underlying the mental processing of topological properties. In the Lord of the Rings routine (Einhorn, 2015), for instance, a ring seems to penetrate a piece of rope tied between the magician’s hands, but the main underlying psychological principle is that we mistake two identical rings for one and the same object, rather than anything that has to do with the mental processing of topological properties. It is also worth noting that my working definition does not refer to the type of magical effect evoked by the tricks. For instance, many tricks can be said to be topological in the sense that the effect is an apparent violation of basic topological laws. Impossible penetrations (Lamont & Wiseman, 2005), such as the aforementioned Lord of the Rings routine, would be topological tricks in this sense, but my definition refers to the underlying psychological principles rather than the nature of the experienced magical effect.

### Mapping Tricks to Underlying Psychological Principles

A basic problem in explaining magic tricks in terms of psychological factors (or any other factors, for that matter) is that most tricks involve several of them. Some of the factors may be sufficient in themselves for creating a magical experience, but often a combination of several psychological factors is necessary. Furthermore, given that the main concern of a conjuror is not to develop detailed and fundamental knowledge of the individual factors, but rather to perform a trick, which is as foolproof as possible, they may often include more factors or ingredients than what is really necessary to create a magical experience, just to be on the safe side. Thus, even if we are aware of one psychological factor contributing to a trick, that factor may not be sufficient for explaining why the trick works so well, and further unknown psychological effects may also be involved. Figuring out what psychological factors are relevant and how they may interact is a difficult problem that can only be resolved through careful experimental dissection of magic tricks. This is beyond the scope of the present article, but I hope that the ideas discussed here may be of value in organizing and inspiring such systematic experimental research.

### The Relationship Between Magic and Imagination

On a general level, it is almost a truism that people fail to discover the secret behind a magic trick because they are unable to imagine it. Indeed, as pointed out by Leddington (2016, p. 260), the experience of magic can be said to “consist in a kind of imaginative failure.” From this point of view, understanding the psychology of magic essentially boils down to understanding why people are unable to imagine the secret method(s) behind the trick.

### Extraneous and Intrinsic Factors That Limit the Imagination

In many cases, it seems reasonable to speak of extraneous factors which block our powers of imagination in the sense that if these factors were removed we would have little trouble imagining the secret method. Consider, for instance, the cigarette trick studied by Kuhn and Tatler (2005). Here, the magician makes a cigarette magically disappear by dropping it into his lap right in front of the spectators’ eyes. Although the cigarette is being dropped openly in full view, the spectators typically fail to notice it due to inattentional blindness (Mack & Rock, 1998) provoked by attentional misdirection (Kuhn & Tatler, 2011). If we were aware of our own inattention, it would probably not be very difficult to imagine that the magician got rid of the cigarette simply by dropping it into his lap. But we are not, due to the well-known failure of visual metacognition (Levin, 2002) associated with change blindness and inattenional blindness. Thus, in this case, our ability to imagine the “secret” move may be said to be restrained by an extraneous factor—our blindness to our own inattentional blindness. In a similar vein, the secret behind the multiplying balls trick—namely, that one of the balls is not a ball at all, but rather an empty semispherical shell—is probably not so hard to imagine in and by itself. Rather, it is probably difficult to imagine because the perceptual phenomenon of amodal volume completion (Ekroll, Mertens, & Wagemans, 2018; Tse, 1999; Van Lier, 1999; Van Lier & Wagemans, 1999) makes it look like a complete ball in a curiously convincing fashion (Ekroll, Sayim, & Wagemans, 2013; Ekroll, Sayim, Van der Hallen, & Wagemans, 2016). Here, our ability to imagine the secret may also be said to be restrained by an extraneous factor, namely, amodal volume completion. Although our ability to imagine the secrets behind magic tricks is often blocked by various extraneous factors such as these (as well as many others), it is also conceivable that our inability to imagine the secrets behind certain tricks is simply due to limitations intrinsic to our faculty of imagery per se. I speculate that the effectiveness of many topological tricks may, at least in part, be due to such intrinsic factors. Accordingly, the analysis of such tricks may teach us something about the intrinsic limitations of imagery.

## Preliminary Explanatory Ideas

I shall now discuss various preliminary hypotheses about psychological factors that may be involved in making the tricks I have described so powerful. Some of these hypotheses are not necessarily mutually exclusive, because several factors may be involved in a given trick. Furthermore, some of my hypotheses are formulated at a more general level than others, meaning that one hypothesis may be a more specific version of another. Table 1 gives an overview of how I envision that the different explanatory principles (top row) can be applied to the tricks described in this study (left-hand column).

**Table 1.** Overview of How the Explanatory Principles Delineated in This Article May Be Applied to the Set of Tricks Discussed.

Trick	Knots as mess	Knots as actions	Attribute substitution	Shape category precedence	Link ownership	Rigidity principle	Simplicity
Perplexing paperclips	–	–	Paperclips as closed loop	Paperclips as closed loop obscures possible v-shape	–	Yes	–
Disappearing knot	Yes	–	Knot as blob	–	–	–	–
Cut and restored rope	Yes	–	Knot as blob	–	–	–	Yes (good continuation)
Houdini shackles	–	Yes	Chain as ring around hands	Frame with external rings obscures frame with internal sliding separators	–	Yes	–
Afghan bands	–	–	Möbius strip as simple loop and cut as a straight plane	–	–	–	–
Handcuffs puzzle	–	Yes	Arms and handcuffs as closed loop	–	–	Yes	–
Hart’s link	–	–	–	–	–	–	Yes (good continuation)
The belt trick	–	–	–	Twist obscures possible loop	–	Yes	–
The twisted band	–	Yes	–	–	–	–	–
The four-dimensional hanks	Yes	Yes	–	Yes	Yes	Yes	–
Jumping rubber band trick	–	Yes	–	–	–	–	–
Wholesale ring removal	–	Yes	Lark’s head knot as single loop	–	–	–	–
Gordian knot	Yes	Yes	Lark’s head knot as single loop	Rope as loop obscures rope as straight and vice versa	Yes	Yes	–
Buttonholed	Yes	–	Shirt as plane with hole instead of as loop	Shirt as plane with hole obscures shirt as loop	Yes	Yes	–
Coin through hole	–	–	Opening as planar hole, coin and hole must be in same plane	Opening as planar hole obscures opening as slit	–	Yes	–

### Attribute Substitution

**Figure 16.**
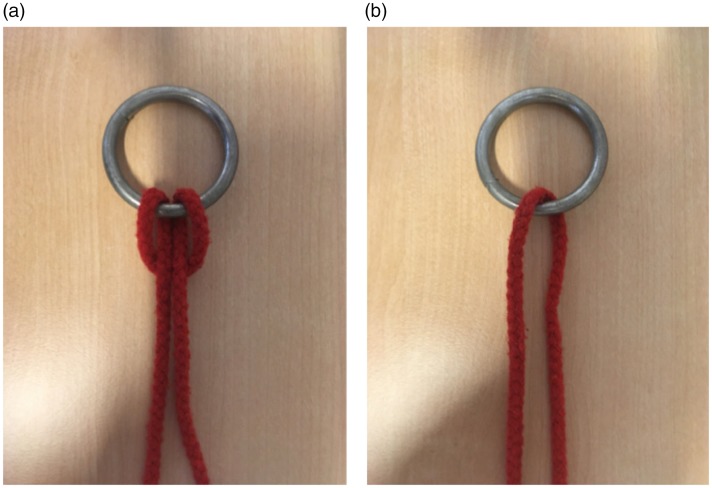
Illustration of a possible attribute substitution in the wholesale ring removal trick. In the actual trick, the lark’s head knot is tied around the ring, and the magician releases the ring from the rope while the spectator has full control over both ends of the rope. This is indeed possible (see Figure 13). If the spectator mentally substitutes the actual arrangement in (a) with the simpler one in (b), however, this is impossible. Thus, this kind of attribute substitution could explain why people experience the trick as magical.

As noted by Kahneman and Frederick (2002, p. 53), “when confronted with a difficult question people often answer an easier one instead, usually without being aware of the substitution.” On a general level, such processes of attribute substitution may be involved in many of the tricks I have described. In the handcuffs puzzle (Figure 7), for instance, the real question of how the actual pair of handcuffed arms may be released from each other may be unconsciously substituted for the simpler question of how two interlinked closed loops (like in Figure 18) may be separated. If the conscious problem-solving process starts on this premise, it is only to be expected that the problem must seem impossible to solve and that it never will be solved. Similarly, if the Möbius loop (Figure 6) is substituted by a simple loop, it would not be surprising that people expect two loops when it is cut along the middle. In this case, it is also conceivable that the path of the cut made by the scissors (or the zipper) along the Möbius band is substituted by a plane intersecting the loop, which would indeed produce two parts. In the case of the cut-and-restored rope trick (see Figure 3), the knot may be substituted by an amorphous blob (in accordance with the knots-as-perceptual-mess-principle, see section “Knots as perceptual mess” described earlier), through which the remaining parts of rope pass and are linked according to the principle of good continuation (Barnhart, 2010; Ekroll et al., 2017; Wertheimer, 1923). In the case of the wholesale ring removal trick (Figure 13), the question of how to release the rope with the lark’s head knot around the ring while the spectators has control over both ends of the rope (Figure 16(a)) may be substituted by the simpler question of how a rope looped around the ring (Figure 16(b)) may be released. If this substitution is made, it would indeed be impossible to find a solution, which would explain why people experience the trick as magical. The same substitution may also account for the Gordian knot trick (Figure 14). In the Houdini shackles routine (Figure 5), the frame and the chains may be substituted with a frame with two closed loops attached to it. An interesting potential case of attribute substitution in magic has previously been discussed by Thomas, Didierjean, and Kuhn (2018).

### The Rigidity Principle: Limits in our Ability to Mentally Simulate Deformations of Objects

One general idea that may explain why the kind of topological tricks described here work so well is that our ability to mentally simulate deformations of flexible objects may be more limited than our ability to mentally simulate the shape-preserving transformations (such as rotations, translations and scalings) that have typically been investigated in research on visual imagery (Carlton & Shepard, 1990; Kirby & Kosslyn, 1990; Shepard & Metzler, 1971). If our mental representations of objects have fewer degrees of freedom than the physical objects they represent, some physically possible transformations should be experienced as impossible. One can think of several reasons why our mental representations might be less well suited for representing deformations than for representing shape-preserving transformations. First, one might expect rigid transformations to be more fundamental, because when an observer moves, seeing an object from different perspectives, the object undergoes a rigid transformation with respect to the observer (Carlton & Shepard, 1990; Gibson, 1966, 1979). Second, rigid transformations are probably more frequent in our environment (Todd, 1982), making it sensible for the perceptual system to exploit rigidity constraints (Koenderink & Van Doorn, 1991; Ullman, 1979). A further reason why we might be poor at imagining the secret behind tricks based on deformable objects is that the set of possible transformations that need to be considered is just much larger when we also allow for nonrigid transformations. Many of the tricks we have considered earlier may, at least in part, be attributable to limits in our ability to mentally simulate deformations of objects. The belt trick (Figure 9) is perhaps most impressive in this regard. Here, it seems to be difficult to imagine that the transformation is possible even when you know how it is done. With other tricks, such as Hart’s link (Figure 8) or the handcuffs puzzle (Figure 7), it is difficult to imagine the transformation before you know the secret but not afterward.

### Prominence of Shape Categories

**Figure 17.**
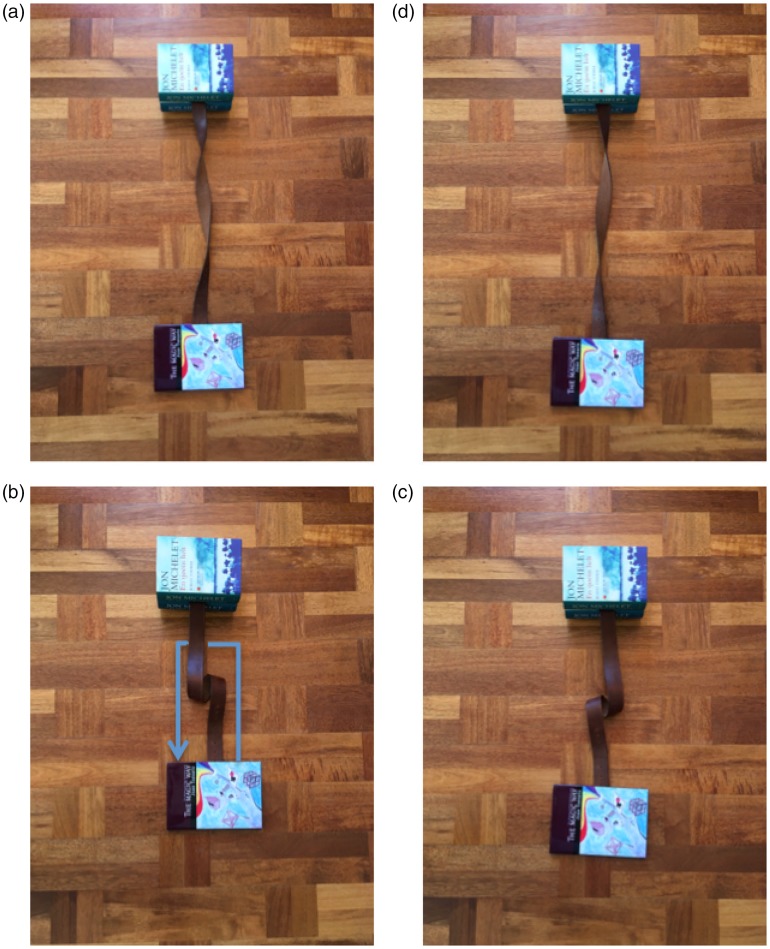
Illustration of a modified version of the belt trick. Rather than transforming an untwisted belt into a belt with a 720° twist (as in the original version, Figure 9), we here transform a 360° clockwise twist (a) into a 360° anticlockwise twist (d), which obviously amounts to the same net amount of twist (720°). Note that the twist in (a) is equivalent to the loop in (b), although it looks very different. This twist is easily turned into the loop simply by releasing the tension of the belt by moving the ends (the books) closer. The downward rightward loop in (b) is transformed into the upward leftward loop in (c) by moving the bottom end of the rope along the path shown by the blue arrow (without ever changing the orientation of the end). Since the latter loop is equivalent to the anticlockwise 360° twist in (d), this simple move has produced a net twist of 720°, just as in the original belt trick.

Obviously, rigid objects have a fixed shape, while the shape of deformable objects may change. One potential reason why we are so poor at imagining possible deformations could be that a reliance on shape or shape categories as a cue to object identity is so deeply entrenched into our mental machinery that it interferes with our ability to imagine deformations which radically change the shape of an object. Consider, for instance, the transformations illustrated in Figure 17, which may be thought of as decomposition of the belt trick (Figure 9) into three simpler parts. Different from the standard form of the belt trick, which starts with an untwisted belt that is twisted by 720°, I here illustrate the equivalent case which starts with a 360° clockwise twist (Figure 17(a)) that is twisted by 720° to obtain a 360° counterclockwise twist (Figure 17(d)). It is not very difficult to imagine that the rightward loop in (b) can be turned into the leftward loop in (c) without ever changing the orientation of the lower end of the belt (held by the book). What seems less obvious, though, is that the twist in (a) is equivalent to the loop in (b) and that the twist in (d) is equivalent to the loop in (c), which means that the movement transforming (b) to (c) actually twists the belt by 720°, as in the belt trick. The configurations of the belt in (a) and (b) look like categorically different shapes, although they have the same topology: (b) can be obtained from (a) simply by moving the ends of the belt a bit closer and letting the belt curl up. The belt configuration in (a) can be continuously changed into at least four perceptually distinct shape categories without changing the orientations of the ends of the belt: In addition to the two shown, the appearance of a helix is possible, as well as an upward looping left-right reversed version of the downward loop shown in (b). As an everyday example of this, many people have probably wondered where all the annoying twists and kinks in an extension cord or a garden hose come from (Jurišić, 1996). The reason is that the loops you made when you coiled it up to store it are turned into twists when you pull it straight. Since the cross-section of the garden hose is round, twists such as in Figure 17(a) are largely invisible, but when the tension is released a bit, the twists tend to turn into loops again.

The division of continuous physical dimensions into discrete perceptual categories is prominent in many domains of perception and cognition (Harnad, 1987; Liberman, Harris, Hoffman, & Griffith, 1957). Hence, it would not come as a surprise if shape categories have an influence on our ability to imagine deformations. According to the old joke, a topologist is someone who cannot tell the difference between a doughnut and a coffee cup, but for us, the problem seems to be that we are too hooked up with shape categories to appreciate the equivalence.

### Link Ownership Principle

**Figure 18.**
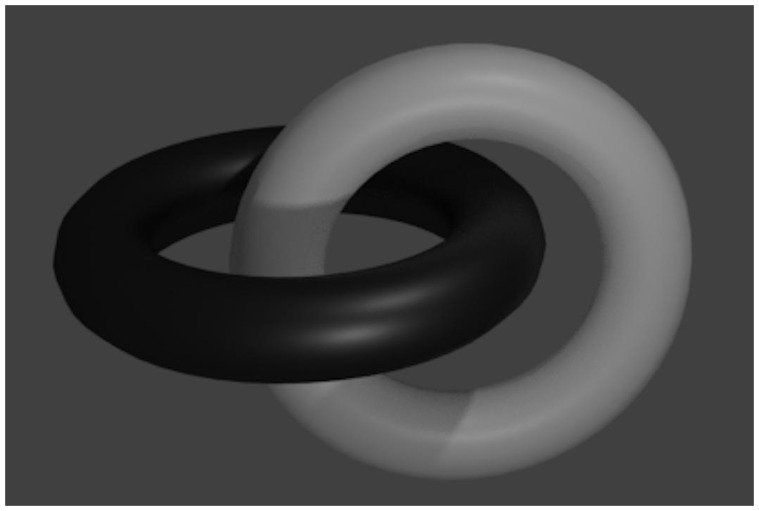
The link between two loops is a shared and mutual property of the two loops. If the white loop is linked to the black one, the black one is also linked to the white one.

**Figure 19.**
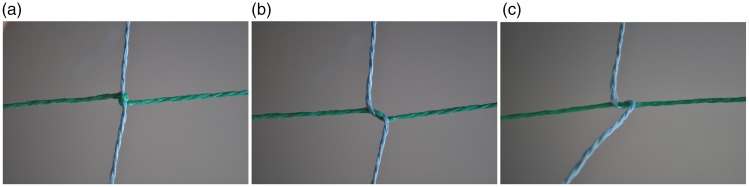
In (a), the horizontal rope is looped once around the vertical one. But topologically speaking, the relation between the two ropes is entirely symmetrical. To see this, just pull a bit at the ends of the horizontal rope, to obtain an intermediate state where the symmetry is obvious (b) or pull it entirely straight (c) to obtain the converse impression (that the vertical rope is looped around the horizontal one).

The link between two loops is a shared and mutual property of the two loops. If the black loop in Figure 18 is linked to the white one, the white one is necessarily also linked to the black one. The same may be said for links between strands of rope. This topological fact is independent of the geometrical configuration of the two components in the link, that is, whether the parts close to the link are curved or straight. However, as illustrated in Figures 14 and 19, we tend to assign ownership of the link to the strand that goes around the other in the current geometric configuration. This phenomenon of “link ownership assignment” is reminiscent of the well-known phenomenon of border ownership assignment in figure-ground perception (Rubin, 1915). A general principle, which would explain both of these phenomena, as well as many others (e.g., Van de Cruys, Wagemans, & Ekroll, 2015), is the principle of exclusive allocation, according to which “a sensory element should not be used in more than one description at a time” (Bregman, 1994, p. 12).

Shape-dependent assignment of link ownership seems to be a powerful factor that makes it difficult to appreciate that the links in the four-dimensional hanks routine (Figure 11), the Gordian knot routine (Figure 14) as well as the buttonholed stunt (Figure 15) are in fact a shared property of the components in the link. Note that this phenomenon of link ownership assignment may be considered a special case of the prominence of shape categories, described in the previous subsection.

### Problems With Establishing and Integrating Frames of Reference

Frames of reference are known to be of considerable importance in human perception at large (Duncker, 1929; Gilchrist et al., 1999), and there is reason to believe that intrinsic frames of reference are used for rigid objects or parts of objects (Hinton & Parsons, 1981; Marr & Nishihara, 1978; Rock, 1973). Establishing and using intrinsic frames of reference for deformable objects, however, are far from straightforward, and some informal observations suggest that we are not very good in using and combining several frames of reference consistently. Consider the twisted rubberband trick (Figure 10). The local 360° rotation illustrated in Figure 10(a) introduces a 360° twist along each of the two vertical strands of the rubber band, as shown in (b). Informal observations suggest that people find it counterintuitive that this 360° twist can be undone by moving the upper hand along the 180° semicircular trajectory shown by the blue arrow in (b), while keeping the orientations of the upper and lower parts of the band level (in the external frame of reference). The reason why the 180° semicircular trajectory corresponds to a 360° twist is that relative to the frame of reference defined by the long strands of the rubber band (which is rotated by 180° between Panels (B) and (C)), the two parts held fixed and level by the fingers are each rotated by 180°, but in opposite directions, thus adding to a rotation of 360°. The observation that people tend to find this counterintuitive suggests that they have trouble integrating and taking the twists from different frames of reference into account. Essentially, the same wrong intuition may be involved in the belt trick (Figure 9). It is probably not too hard to realize that the motion of the end of the belt that untwists the 720° twist in Figure 9(a) (although the orientation of the end never changes) involves a 360° path of the end moving around a part of the belt itself (or vice versa, of course), such that the untwisting of a single 360° twist may not appear all that mysterious. But it may be less obvious that the very act of keeping the orientation of the end fixed at all times adds a second full twist, in much the same way as the act of keeping both ends level in the twisted rubber band trick while turning the main axis by 180° corresponds to a 360° twist.

### Knots as Actions

Many of the tricks we have considered suggest that our representations of even moderately complicated spatial objects like simple knots are of very limited fidelity (see section “Knots as perceptual mess”) and that our ability to mentally simulate possible deformations of objects is severely limited (see section “The rigidity principle: Limits in our ability to mentally simulate deformations of objects”). The many counterintuitive effects used in these tricks suggest that whatever visual imagery we engage in when trying to understand these tricks and phenomena cannot be “mental analogs” (or “dynamic visual images”) of the objects and their inherent flexibility. The deceptive properties of some of the tricks are easier to understand if we assume that the observers represent the configurations of flexible objects as actions rather than literal analogs of the physical objects (see Casati, 2013, for a related idea).

Consider, for instance, the wholesale ring removal trick (Figure 13). If the knot tied around the ring is mentally represented in terms of the actions made to tie it, and untying it simply as the reverse sequence of actions, it is easy to see why the spectators experience it as impossible to untie the knot: Since the loose ends originally tied around the ring are held by the spectator, the knot cannot be untied by reversing the original sequence of actions. Thus, the solution, which is to untie it by performing a different sequence of actions, is simply not an option in terms of the spectators’ mental representation of the knot. Clearly, this explanation can also be applied directly to the Gordian knot routine (Figure 14).

This “knots-as-actions hypothesis” also accounts well for the deceptive power of the jumping rubber band trick (Figure 12). In this case, the action the magician uses to get his index and middle finger out of the rubber band is not the simple reversal how they got in there. Rather, the fingers fold back such that the fingertips leave the loop first.

This hypothesis may also contribute to the deceptive power of the four-dimensional hanks routine (Figure 11). Here, the first loop tied around the vertical rope (Figure 11(a) and (b)) is cancelled by performing a different action (tying a loop around the horizontal rope) rather than by simply reversing the first action.

In general, the technique of adding further knots or loops, seemingly complicating the initial knot but actually untying it, is used in a large number of magical tricks.

### Simplicity Principle and the Hidden Inverse

In many magic tricks, a seemingly solid knot (or collection of loops) is easily released due to a second knot, which is the exact opposite or “inverse” of the first, and thus neutralizes it. Judah’s penetration trick (see Gardner, 1958) is a good example of this. The principle of two mirror-image loops neutralizing each other is also employed in Hart’s link (Figure 8), but here, the link the spectators believe has been formed was never even created because it was simultaneously neutralized by a mirror-image “link” that was created at the same time but remained hidden behind the conjurer’s head. Why this possibility mostly fails to enter peoples mind is not entirely clear, but a strong preference for the simplest possible interpretation (Van der Helm, 2014) of the visible parts (on a perceptual, not conscious conceptual level) and the principle of good continuation (Barnhart, 2010; Ekroll et al., 2017; Wertheimer, 1923) could be the driving force here.

## Summary and Conclusions

In this study, I have described a selection of surprisingly powerful magic tricks, which can be loosely categorized as topological in the sense that they involve the use of flexible materials. Although it seems difficult to explain why these tricks are so powerful and why they evoke the experience of magic (i.e., impossibility), I have delineated some preliminary ideas about important psychological principles involved, namely:

- *Mess principle:* Even moderately complicated spatial objects like simple knots are not perceptually organized, they are merely represented as amorphous blobs and we lack an immediate visual understanding of them. Understanding even simple knots requires effortful and time-consuming serial mental curve tracing.
- *Attribute substitution:* Even moderately simple spatial configurations are mentally substituted by much cruder mental representations.
- *Rigidity principle:* Many bendable objects are more flexible than our mental representations of them.
- *Prominence of shape categories:* The current geometrical shape category of a flexible object is very prominent in our immediate visual experience and may block imagery of alternative possible shape categories the object may assume.
- *Link ownership principle:* Links between two strands of rope are mutual and symmetric in a topological sense, and therefore belong to both strands, but we tend to assign ownership of the link only to the strand that goes around the other in the current geometric configuration.
- *Frames of reference principle:* It is particularly difficult to establish a single and unique internal frame of reference for flexible objects. Spatial reasoning about flexible objects may require the establishment of several local intrinsic frames of reference, which may be difficult to integrate and keep track of.
- *Action principle:* Knots are encoded as actions. We falsely assume that the only way to untie them is to reverse the original tying action.
- *Simplicity principle:* A strong preference for the simplest possible interpretation of the visible parts of an object may severely limit imagery for more complicated possibilities.

These principles describe heuristics, limitations, and biases of our cognitive system that sometimes lead to errors of perception and imagery. Obviously, such errors are necessary for creating magical experiences, but they are not sufficient. Simply being deceived does not in itself create an experience of magic. It has been argued that some failure of metacognition is a necessary causal factor in the creation of a magical experience (Ekroll et al., 2017; Kuhn, 2019; Kuhn et al., 2014). Tricks based on inattentional blindness, for instance, would not create an experience of magic if our immediate perceptual experience would reflect the fact that we are essentially blind at the location where the secret move happens in full view (Kuhn, 2019; Kuhn & Tatler, 2005; Ortega et al., 2018). Clearly, if we are blind in a region of the visual field and aware of it, it is obvious that the magician is free to do just about anything there without us noticing. Thus, the *conditio sine qua non* in tricks based on inattentional blindness is the failure of visual metacognition associated with inattentional blindness—the illusion that we perceive everything in front of our eyes in clear detail (Levin, 2002). Applied to the tricks discussed in this article, it therefore seems reasonable to speculate that analogous failures of metacognition are essential for creating the experience of magic. In the section “Knots as perceptual mess,” I proposed that the tricks discussed there involve a kind of “topology blindness” or “mess principle,” where the perceptual system fails to perceptually organize knots into well-defined 3D objects and that our failure to experience this topology blindness explain why these tricks evoke a feeling of magic (impossibility) rather than just a feeling of not seeing the knots very well. That is, when we look at the world, some things are perceptually organized and others are not, but this difference is not reflected in our immediate visual awareness. Some of the tricks discussed in the section “Illusions and limitations in visual imagery and mental simulations” seem to involve limitations in our ability to mentally simulate or imagine deformations of objects, as if our mental representations of deformable objects have fewer degrees of freedom than the objects they represent. Importantly, though, this inflexibility of our imagery is not reflected in our phenomenal experience. We feel unlimited in our freedom to imagine deformations of objects, but the deceptive power of these tricks strongly suggest that we are not. This failure of imagery meta-cognition (or “meta-imagery”) would explain why these tricks evoke a sense of magic (impossibility) rather than just a sense of not being able to imagine how things can deform. This idea is in line with Pylyshyn’s (1973, 2003b) observations about the misleading phenomenology of imagery. Furthermore, a central idea of attribute substitution is that we are not aware of the substitution taking place (Kahneman & Frederick, 2002).

A potentially interesting general observation pertaining to the set of tricks discussed in this study is that some of them seem to retain a certain residual magic quality even when the spectator knows what is going on (or the correct way to think about what is going on). In the case of The Afghan Bands, for instance, a correct intuition that explains why the cutting of the Möbius band must produce a single long loop can be developed by thinking of the original uncut band as two separate Strands A and B to begin with. When the 180° twist is introduced before joining the ends, one end of Strand A is connected to one end of Strand B and the other end of Strand A is connected to the other end of Strand B, making it obvious that a single long strand must indeed result. But even when one is aware of this correct intuition, the misleading intuition that two separate loops must result seem does not go away but seem to persist and coexist in a curious manner together with the correct intuition. Similarly, developing a correct intuition of what is going on in The Belt Trick also does not seem to make the misleading intuition go away. In the case of other tricks, however, such as The Handcuffs Puzzle or Hart’s link, no misleading intuitions seem to persist once the secret is known. Ekroll et al. (2017) have previously noted that tricks based on amodal completion seem to retain a residual magic quality even when the spectator knows the secret behind the trick. In the case of amodal completion, we attributed this residual magic quality to a perceptual illusion that persists in spite of conflicting conscious knowledge. On this note, one may speculate that the residual magic quality of some of the tricks discussed in this study also indicate that perception-like processes are involved. Thus, further empirical study of these informal observations regarding the presence or absence residual magical experiences in topological tricks seem potentially rewarding.

Some of the psychological principles I have discussed, such as attribute substitution, are well known in cognitive science, while others, such as the action principle and the link ownership principle, are novel. I believe that these principles are of relatively broad applicability but the list is probably not exhaustive, and I believe that further analysis of topological magic tricks and puzzles will reveal further new insights into perception, imagery, and reasoning.

Magic tricks can be likened to Gestalt demonstrations in the sense that although they do not obviate the need for careful experimentation, they are helpful in putting phenomena on the agenda that are so powerful that almost everybody will experience them. The very fact that something is a magic trick already suggests that we are dealing with a robust and powerful phenomenon, and therefore, the study of magic may drive cognitive science forward in much the same way as Gestalt demonstrations have done so.

The tricks discussed in this article highlight quite stunning limitations in our perception of and imagery about topological properties. It is worth pointing out, however, that there is a large literature suggesting that our visual system is more sensitive to topological structure than geometrical structure, and that coding of topological relationships plays a fundamental role in visual perception (Barth, Ferraro, & Zetzsche, 2001; Casati, 2009; Chen, 1985, 1990, 2001; Chen, Zhang, & Srinivasan, 2003; Chien et al., 2012; Han, Humphreys, & Chen, 1999; Hecht & Bader, 1998; Huang, Huang, Tan, & Tao, 2009; Pomerantz, 2003; Todd, Chen, & Norman, 1998; Zhou, Luo, Zhou, Zhuo, & Chen, 2010). Thus, an important question for future research is to figure out what mechanisms and heuristics the perceptual system uses for coding topological properties, and how these mechanisms and heuristics may lead to superior performance in some cases and systematic errors in others (Ullman, 1984).

As pointed out by Parsons (1995),

Scientific understanding of which spatial transformations can be performed readily and accurately and which are performed poorly is important for various reasons. It can guide inferences about the processes and representations underlying human spatial transformations in imagination, spatial reasoning, perception, and motor behavior. In addition, a detailed characterization of this capacity will have implications for applied areas such as design, human-computer interaction, and the practice and teaching of mathematics, physics, and engineering. (p. 1259)

It appears reasonable to assume that systematic theoretical and experimental analysis of the many illusions of imagery evident in magic tricks and puzzles could be of great value in this endeavor.

## Notes

1 Alternatively, he may make the knot magically disappear by grasping it in his hand and moving it along the rope until he reaches the end of it. The knot seems to have magically been dragged off the rope.

2 Bearing in mind that a donut is topologically equivalent to a coffee cup, this trick can of course also be performed with the latter.

3 See https://www.youtube.com/watch?v=IMORqoCNybY.
